# The (In)significance of Executive Functions for the Trait of Self-Control: A Psychometric Study

**DOI:** 10.3389/fpsyg.2018.01139

**Published:** 2018-07-09

**Authors:** Edward Nęcka, Aleksandra Gruszka, Jarosław Orzechowski, Michał Nowak, Natalia Wójcik

**Affiliations:** Institute of Psychology, Jagiellonian University, Kraków, Poland

**Keywords:** self-control, self-regulation, executive functions, cognitive control, intelligence

## Abstract

Self-control (SC) is an individual trait defined as the ability to pursue long-distance goals in spite of the obstacles generated by current desires, innate or learned automatisms, and physiological needs of an organism. This trait is relatively stable across the life span and it predicts such important features as level of income, quality of social relationships, and proneness to addictions. It is widely believed that the cognitive substrate of SC involves the executive functions (EFs), such as inhibitory control, shifting of attention, and working memory updating. However, the empirical evidence concerning the relationships between trait SC and EFs is not convincing. The present study aims to address two questions: (1) what is the strength of relationships between trait SC and EFs, and (2) which aspects of SC are predicted by particular EFs, if at all. In order to answer these questions, we carried out a psychometric study with 296 participants (133 men and 163 women, mean age 23.31, SD 3.64), whom we investigated with three types of tools: (1) a battery SC scales and inventories, (2) a battery of EFs tasks, and (3) two general intelligence tests. Structural equation modeling approach was used to analyze the data. We found that the latent variables representing SC and the latent variable representing EFs did not show any relationship. The standardized path coefficient between EFs and general intelligence turned out rather strong. We conclude that the trait of SC, measured with questionnaires, does not depend on the strength of cognitive control, measured with EFs tasks.

## Introduction

Self-control (SC) is a human capability to pursue distant valuable goals in spite of obstacles produced by situational influences, innate or learned automatisms, and inner impulses caused by current physiological needs. Traditionally, this phenomenon has been explored within two research paradigms. Firstly, there are studies publishes by Roy F. Baumeister and his colleagues, who showed that doing a task that requires effortful control results in transient reduction of one’s capability to exert SC furthermore (Muraven and Baumeister, 2000). For instance, watching a movie with an instruction to ignore subtitles appearing at the bottom of the screen makes a person less able to do higher-order cognitive tasks, such as cognitive tests (Baumeister et al., 1998). Such studies provided empirical background for the so-called strength theory of SC, also known as the ‘ego depletion’ theory (Baumeister et al., 2007; Hagger et al., 2010), according to which self-regulation is a kind of resource that can be ‘spent’ on tasks requiring effortful control. The more is ‘spent’ on a preceding task the less can be ‘spent’ on the following tasks, unless the resources are renewed during a recreational break. The ‘ego depletion’ effect, which we suggest to label with a neutral term ‘the Baumeister effect,’ is now debated concerning its strength and generality (Lurquin et al., 2016). Secondly, there are studies published by Mischel (1974) and his collaborators (Mischel et al., 1989), showing high predictive value of one’s ability to refuse immediate gratification for the sake for a much larger but delayed reward. In the so-called marshmallow paradigm, preschool children were rewarded with one cake, which they could eat immediately, unless they decided to wait for the second cake, which – unbeknownst to them – would be delivered 15 min later. The median waiting time for the second cake was about 7 min, although some children could not wait longer than 1 min whereas others could withstand the whole waiting period. These huge individual differences in the ability to delay gratification, measured in the preschool period, appeared highly predictive concerning important aspects of adult life, such as higher income, better and more stable relationships, and reduced vulnerability to addictions (Mischel et al., 1988; Casey et al., 2011).

Recent approaches to SC underline its involvement in the process of value-based decision-making (Inzlicht and Berkman, 2015; Berkman et al., 2017). The decision to exert SC, or not, is described as a function of choice, determined by different values ascribed to potential personal goals. According to this account, sometimes people are able to delay gratification because the value of the delayed goal is much higher than the value of the immediately accessible goal, although the latter looks rather tempting and may be reached without any effort. In other cases, people with enough resources to control themselves may decide that immediate pleasure is more valuable than a long-distance goal, whose attainment needs time and effort. The ‘Baumeister effect’ can therefore be accounted for in terms of value weighting and failures of motivation rather than in terms of depletion of ‘ego resources.’

In this paper, we discuss the problem of cognitive underpinnings of SC, understood as a relatively stable individual trait. We assume that such a trait can be assessed with reliable psychometric tools, and the scores gained by a person with such tools can be related to other individual traits, such as personality and intelligence. Next, we assume that the trait of SC is subserved by specialized cognitive functions, similarly to other individual traits. For instance, it has been convincingly demonstrated that general fluid intelligence depends on individual differences in working memory capacity (e.g., Colom et al., 2004; Chuderski and Necka, 2012, and the trait of creativity is related to divergent thinking skills (McCrae, 1987; Baer, 1993). Since stable traits are hardly susceptible to experimental manipulations, the studies on cognitive underpinnings of individual differences are mostly correlational in nature, so the causal explanations are quite risky. It may be claimed, for example, that capacity of working memory determines the level of general fluid intelligence or that the level of general intelligence determines accuracy in dealing with working memory tasks. The former account is sometimes referred to as the bottom-up approach (cognitive functions determine the general trait), whereas the latter one is called the top-down approach (the general trait determines cognitive functions). The training studies (e.g., Jaeggi et al., 2008) showed that enhancement of intelligence may result from systematic improvement of working memory capacity (a far transfer effect), which favors the bottom-up stance. The bottom-up explanations, according to which specific cognitive functions determine the level of intelligence, rather than the opposite, are also supported by theoretical considerations (e.g., Sternberg, 2008). However, there are serious doubts concerning the question whether intelligence really can be improved through training (e.g., Shipstead et al., 2012), so the bottom-up explanations of intelligence still need stronger empirical evidence.

As regards the trait of SC, there is a widespread conviction that it is cognitively subserved by executive functions (EFs). According to a definition proposed by Akira Miyake and his co-workers, EFs are ‘…general-purpose control mechanisms that modulate the operation of various cognitive subprocesses and thereby regulate the dynamics of human cognition’ (Miyake et al., 2000, p. 50). Various cognitive processes, involved in reception and storage of information (perception, memory), but also implicated in manipulation with mental representations (thinking), need some kind of integration and supervision. Without such a management, human cognition would get disintegrated, thus being unable to play its fundamental function, namely, the control of behavior. In other words, cognitive processes must be effectively controlled in order to be able to command our behavior (Diamond, 2013). Cognitive control seems particularly important in situations that need overriding automatic behavioral tendencies, since such situations are very complex, unexpected, or novel. In such situations, a dominant behavioral tendency must be suppressed (inhibition), a new pattern of behavior or a new mental set must be initiated (shifting), and the awareness concerning the ongoing task must be refreshed (working memory updating). No wonder, then, that Miyake and co-workers consider Inhibition, Shifting, and Updating, to be the most important EFs.

The definition proposed Miyake et al. (2000) declares that EFs are general-purpose mechanisms, meaning that they should be implicated in all kinds of cognition. However, they appeared particularly important for higher-order cognitive processes, such as thinking and problem solving. Consequently, EFs must be considered as important determinants of individual differences in cognition. Indeed, the results of research on intelligence (e.g., Nęcka, 1998; Chuderski and Nęcka, 2010; Cole et al., 2012) and creativity (e.g., Groborz and Nęcka, 2003; Benedek et al., 2014) support this conclusion. Regarding SC, many authors seem to be convinced that EFs demonstrate huge individual differences that subserve individual level of SC (e.g., Hofmann et al., 2009, 2012). For instance, Kotabe and Hofmann (2015, p. 625) maintain that ‘the importance of EFs to SC is clear.’ In the theoretical model outlined by these authors, individual differences in SC depend, among other cognitive and motivational factors, on the capacity to exert control. This capacity is supposed to be measured by tasks that engage executive control.

On the one hand, the importance of EFs for SC has been demonstrated in many studies. For instance, the longitudinal study carried out by Friedman et al. (2011) showed in that preschool children who were able to restrain themselves from immediate gratification demonstrated, as adolescents, higher level of the common EF factor (closely related to Inhibition) and Shifting, but not Updating. Young et al. (2009) determined that behavioral misconduct among adolescents (e.g., substance abuse) was correlated with lower scores in three EFs tasks: Stroop, anti-saccade, and stop-signal. People who were able to delay gratification in the marshmallow experiment at the age of four showed better performance in the ‘go-no go’ and prepotent response inhibition tasks at the age of 16–18 (Eigsti et al., 2006). There are also findings suggesting that criminal and violent behavior may be related to deficient executive control (Meijers et al., 2015).

On the other hand, there are studies showing very weak relationships between EF tasks performance and self-report measures of behavioral control (Nęcka et al., 2012). Duckworth and Kern (2011) carried out a meta-analytic study (282 samples, 34,564 participants), trying to establish the strength of relationships between various measures of SC (self-report, informant report, delay of gratification) and executive control. The authors found rather weak inter-correlations *between* various types of tasks, but also *within* each type of tasks. For instance, EF tasks appeared inter-correlated among themselves at the level of *r* = 0.14; the average correlations between EF tasks and other groups of measures appeared weak as well: *r* = 0.11 for delay tasks, *r* = 0.10 for self-reports, and *r* = 0.14 for informant reports. Interestingly, average convergent validity measures appeared much higher for self-report (*r* = 0.48) and informant-report (*r* = 0.54) measures. These results suggest that both SC and executive control are highly heterogeneous constructs that need to be assessed with heterogeneous batteries of tests, scales, or questionnaires. They also suggest that the category of EF tasks is much more diversified than the category of self-report and informant-report measures. Low level of inter-correlations between various EF tasks may result from their ‘impurity,’ meaning that such tasks measure not only one specific EF but also other functions, not to mention a number of other factors, such as general speed of responding, attentional alertness, susceptibility to boredom during long experimental sessions, lack of computer phobia, etc.

In this paper, we attempt to investigate the relationship between executive control, measured with standard EF tasks, and SC, measured with both self-report and informant-report questionnaires. In order to overcome to problem of diversity and ‘impurity’ of EF tasks, we adopted the structural equation modeling approach with a relatively large sample of participants. The SEM approach allows extraction of latent variables that ignore specificity of various tasks, thus expressing the common factor that these tasks refer to. Additionally, we included two general intelligence tests, in order to establish whether possible relationships between SC and EFs would be moderated by the general mental ability, which is implicated in executive control as well.

## Materials and Methods

### Participants

We investigated 296 participants recruited via two social media networks. There were 133 men and 163 women in the sample. Their mean age was 23.31 years (*SD* = 3.64). All participants were from outside of the Psychology Department. Participants obtained 60 PLN (ca. 15 €) for 4 h of testing, including a 15-min refreshment break.

### Ethics Statement

The committee for ethics in studies involving human participants, assigned by the Institute of Psychology, Jagiellonian University in Krakow, approved this study on the basis of extended description of methods, materials, and procedure. According to the Helsinki declaration, participants signed written informed consent forms.

### Self-Control Measures

#### NAS-50

This is a self-report questionnaire of SC developed by us (Nęcka et al., 2016). It consists of 50 items divided into five subscales: Initiative and Persistence (IP), Proactive Control (PC), Switching and Flexibility (SF), Inhibition and Adjournment (IA), and Goal Maintenance (GM). This tool has been subjected to the validation study with 934 participants (see: Nęcka et al., 2016). Its reliability was assessed with internal consistency measures (Cronbach’s α = 0.86) and test/retest approach (intraclass correlation coefficient ICC = 0.94). The validation study revealed that five subscales correlated with the NAS-50 general score at the moderate or high level (+0.47 < *r* < +0.70, depending on the subscale). The general score turned out strongly associated with Baumeister’s (see: Tangney et al., 2004) SC Scale (*r* = 0.77). Also, the Conscientiousness dimension of the Big Five model predicted the NAS-50 general score (*r* = 0.54). So, the convergent validity of NAS-50 seems suitable. As to divergent validity, this measure appeared completely independent of general mental ability scores (see: Nęcka et al., 2016, for details).

#### NAS-40

This is a mutation of NAS-50 prepared for the informant report studies. We removed 10 items from the original version (NAS-50), due to their overly introspective content that would make them difficult to use by informants. The remaining 40 items were converted into the third person grammatical version (e.g., ‘He/she is usually not late for meetings’ instead ‘I’m usually not late for meetings’). In this way, NAS-40 became possible to fill in by somebody who knows the participant proper (a colleague, a teacher, etc.). The reliability measures of NAS-40 turned out to be satisfactory (α = 0.84, ICC = 0.92).

#### Self-Control Scale (SCS)

The Self-Control Scale (SCS), developed by Tangney et al. (2004), is a self-report questionnaire consisting of 36 items. The authors report good reliability characteristics (Cronbach’s α = 0.89, test–retest reliability = 0.89).

#### Conscientiousness (C)

We administered the NEO-FFI questionnaire (Costa and McCrae, 1992) in the Polish adaptation (Zawadzki et al., 1998). This tool was important for its Conscientiousness scale, since description of this personality dimension pertains to some aspects of SC, understood as an individual trait.

### Executive Control Tasks

We administered a battery of five computerized EF tasks that were supposed to engage three major EFs: Inhibition, Shifting, and Updating (Miyake et al., 2000). For Inhibition, we selected the Stop signal task. For Shifting, we chose the CATT procedure, already used in some studies of ours (Nęcka et al., 2012). For Updating, we decided on the n-back procedure, which requires constant refreshment of the content of working memory. Additionally, this specific version of n-back requires that false signals (a.k.a. ‘lures’) be ignored, so this task allows assessment of the Inhibition function as well. The second task engaging the function of Updating is called COUNT, since it requires mental counting of sequentially presented stimuli up to their third appearance and again from the beginning. Furthermore, we administered the Stroop task, although it is hard to decide which specific EF this task refers to. However, in spite of its ‘impurity’ it is widely used in the cognitive control research as an example of the category of interference resolution tasks (Chuderski et al., 2012). It is supposed to capture the Inhibition function as well (Miyake et al., 2000). All these tasks have been already used in many studies carried out in our lab (e.g., Chuderski and Nęcka, 2010; Chuderski and Necka, 2012; Chuderski et al., 2012; Nęcka et al., 2012). Ideally, it would be advisable to have at least two tasks engaging each EF, and this was our initial plan. However, we could not find an acceptable version of a second task that would involve the function of Shifting, so we decided to use only the CATT procedure. The function of Updating is represented by two tasks: COUNT and n-back, the latter being important in reference to signal detection only. The function of Inhibition is represented by Stop-Signal, Stroop, and n-back again, the latter being important as far as inhibition of distracting lures is concerned.

#### Stop Signal

Participants performed the stop signal task (Logan, 1994) modified by Verbruggen et al. (2008). Pictures of an arrow heading left or right served as the visual stimuli in this version of SST. Participants were asked to press left or right arrow keys according to direction of the arrow on the screen. These *go* stimuli were presented randomly one at a time, each with 50% probability. Participants were supposed to be as fast and correct as possible unless an auditory stop signal was presented over the headphones. In this case they were instructed to stop the response. After successful inhibition, the interval between *go* and *stop* stimuli became 50 ms longer, after unsuccessful inhibition the interval became 50 ms shorter (minimum 50 ms, maximum 1150 ms). The stop-signal delay (SSD) was set to 250 ms at the start of each experimental block. This task allows calculation the SSRT (stop signal reaction time), according to the following rationale: SSRT is the time elapsing between the signal of stop and the internal (i.e., mental) reaction to this signal. If there is a.50 probability of responding in spite of the stop signal, time of the unobserved internal response to the signal of stop must be equal to the mean reaction time for go responses. Since SSD is adjusted on the basis of accuracy observed in the recent trial, the probability of responding in spite of the signal of stop must be 0.50. Therefore, SSRT is calculated as the difference between mean RT and adjusted SSD (see: Verbruggen et al., 2008). The shorter (faster) the SSRT the better is one’s ability to inhibit the unnecessary response.

#### N-Back

We used the figural version of the n-back task, the same as in Experiment 5 reported by Chuderski and Necka (2012). The task consisted in serial presentation of simple figural symbols, such a star, a triangle, an arrow etc., each approximately 2.5 cm × 2.5 cm in size. Stimuli remained at the screen for 1500 ms and were masked for 300 ms. The task consisted of four series. In every series we presented 88 stimuli, so altogether there were 352 stimuli showed to each participant, plus some training stimuli before each series. Sixteen out of 88 stimuli in every series were presented twice. The participants were supposed to decide whether the second presentation took place *n* elements after the first one. The predefined *n* number equaled two. Hence, participants were instructed to press a space bar if and only if the currently presented symbol had already appeared two items back. For instance, if a symbol reappeared in the stream of stimuli separated by just one other symbol (e.g., star, triangle, star again) this repeated symbol became a target that required detection and speedy response with the space bar. If an item reappeared too early, i.e., immediately after its first presentation, or too late, i.e., separated by two symbols instead of just one, it was to be ignored. Stimuli reappearing too early (*n* = 1) or too late (*n* = 3) were classified as ‘lures,’ since their function was to ‘tempt’ participants to respond with no required accuracy. There were eight targets, four *n* = 1 lures, and four *n* = 3 lures in every series. Majority of stimuli (72 in every series) did not reappear shortly after their first presentation. These stimuli may be termed “noise,” since they were to be ignored. If a participant responded to such stimuli, he/she committed the error of false alarm. Also, if a participant pressed the space bar in response to the stimuli that reappeared at “wrong” positions, i.e., *n* = 1 or *n* = 3, he/she earned the error of lure detection. We registered accuracy scores for each participant, defined as the proportion of correct signal detections and the proportion of erroneous lure detection. We also registered reaction time of every response.

#### COUNT

This task was based on the mental counters procedure (Larson, 1986). Participants were presented with a sequence of randomly repeated figures: triangle, circle or square. They were supposed to count how many times each figure has already appeared. If the currently displayed figure appeared for the third time in the sequence, the participants had to press space key. Additionally, the third appearance of any figure meant that counting of this particular type of stimulus should start from the beginning. In this way, the participants had to keep in mind and update three ‘stacks’ of elements (i.e., figures). Auditory feedback took place after each erroneous reaction or lack of reaction for the third presentation of any figure. There were 45 instances of the third repetition (15 for each figure). The program registered the number of misses (lack of notification of the third repetition), the number of other errors, and the mean response time.

#### CATT

This task allows the analysis of controlled switching of attention and its logic was borrowed from Meiran (1996). Participants were presented with separate digits, which appeared at the screen for 3 s or until the response was made. They were instructed either to categorize the digits into odd (left key) and even (right key) or to categorize them into smaller than five (left key) and bigger than five (right key). Of course, the digit “5” had to be removed from the set of stimuli. Given that the task required double categorization, the participants were provided with cues that indicated which task they should fulfill in the upcoming trial. The cues were just single words followed by a question mark, i.e., “EVEN?” or “SMALLER?”, and they appeared 500 ms before the stimulus proper. Participants were trained first in the correct use of instructions, response keys, and cues (20 trials, 1000 ms for a cue). Then, they were asked to perform a series of 148 trials, which were arranged randomly in sequence in relation to repeat and switch conditions. Participants had 3000 ms to respond (4000 ms in the training phase). Each digit that served as a stimulus was masked for 500 ms (1000 ms in the training phase). We registered the reaction time of correct responses as well as misses and false alarms. Participants were asked to be accurate rather than quick.

#### Stroop

We used the numerical version of the Stroop task, which required counting digits and ignoring their meaning (Fox et al., 1971; Chuderski et al., 2012). The screen showed three, four, five, or six exemplars of a digit drawn from the set [3, 4, 5, 6]. Each digit was 0.6 cm × 0.8 cm in size. In congruent trials, the number of stimuli was in concord with the digits to be counted (e.g., four exemplars of the digit ‘4’). In incongruent trials, the former and the latter differed (e.g., five exemplars of the digit ‘4’). Trials lasted 3 s or until response was given. There was also a neutral condition, in which participants were supposed to respond to the number of stimuli not being digits. The instruction was to avoid reading a digit and to press a response key that was assigned to a presented number of stimuli. There were 60 congruent, 60 incongruent, and 60 neutral stimuli altogether. Accuracy and latency of each response was registered. Dependent variables (DVs) were as follows: the number of correct responses in each condition and the average response time in each condition.

### Intelligence Tests

#### Raven

In order to assess their level of fluid intelligence, participants were given Raven’s Progressive Matrices Advanced Version (Raven et al., 1983) in the paper-and-pencil form. This test consists of 36 items that include a three-by-three matrix of figural patterns. The bottom-left pattern is always missing. A testee is supposed to fill in this blank space with the correct pattern, which he/she can choose from eight response options provided at the bottom of the sheet. This test is regarded a good estimation of general fluid intelligence since it requires grasping the abstract rules that govern the composition of the matrix and to apply this rule while choosing the accurate response. The pilot study showed that the whole procedure was too long and tiresome for participants; therefore, only the even items from RAPM were administered, which did not worsen the reliability of assessment.

#### Analogies (TAO)

We also administered another paper-and-pencil test of fluid reasoning, which requires understanding and using the relations of analogy. Jarosław Orzechowski and Adam Chuderski have designed this analogical reasoning test in our lab. It has been used in several published studies (e.g., Chuderski and Necka, 2012; Chuderski et al., 2012). The test includes 36 figural analogies in the form ‘A is to B as C is to X,’ where A, B, and C are types of relatively simple patterns of figures, A is related to B according to two, three, four, or five latent rules (e.g., symmetry, rotation, change in size, color, thickness, number of objects, etc.), and X is an empty space. The task is to choose one figure out of four choice alternatives that relates to figure C, as B relates to A. Again, only the even items of TAO were administered.

### Procedure

Participants were invited to the lab in pairs. In the ads that advertised participation in this study, we put the precondition that two people are welcome together if they know each other for at least 6 months. This requirement was important, since every participant was supposed to fill in all self-report questionnaires plus one informant-report tool (i.e., NAS-40), pertaining to the colleague he/she appeared with. After reporting to the lab, participants filled in the conscious consent form, and next they started to do the proper tasks in the following sequence: NAS-50, NAS-40, SCS, CATT, Count, n-back, Stroop, Stop signal, NEO-FFI, Raven, TAO. In the middle of the procedure, which took ca. 4 h altogether, participants had a 15-min break when they could have snacks and soft drinks.

## Results

In **Table 1** we report basic descriptive statistics. Since every computerized EF task yielded several indices, such as latencies and (in)accuracy measures for different series or level of difficulty, we do not report all possible measurement outcomes. Only the DVs that entered into further structural modeling are displayed in **Table 1**. These data pertaining to range and standard deviation suggest that there were huge inter-individual differences among participants, which make further correlational and structural analyses acceptable.

**Table 1:** Descriptive statistics.

	Minimum	Maximum	Mean		Standard deviation
			Statistic	Standard error	
Age	18.00	43.00	23.35	0.21	3.64
NAS-50	105.00	225.00	165.15	1.17	20.04
NAS-40	85.00	180.00	136.42	1.01	17.45
S-C Scale	65.00	164.00	110.71	1.02	17.57
C (NEO-FFI)	11.00	52.00	32.96	0.46	7.89
TAO	0.00	18.00	13.93	0.18	3.07
RAPM	1.00	23.00	16.16	0.22	3.76
CATT (errors)	0.00	99.00	12.32	1.00	17.28
COUNT (errors)	0.00	33.00	11.56	0.42	7.20
SST (Ssrt)	130.16	477.85	246.73	2.72	46.86
N-BACK (correct)	6.00	24.00	17.72	0.22	3.84
N-BACK (lures)	0.00	21.00	4.04	0.22	3.72

*NAS-50, Self-control questionnaire, self-report version (Nęcka et al., 2016). NAS-40, Self-control questionnaire, informant version (Nęcka et al., 2016). SCS, Self-Control Scale (Tangney et al., 2004). C (NEO-FFI), Conscientiousness from the NEO-FFI questionnaire. TAO, Analogical Reasoning Test. RAPM, Raven’s Advanced Progressive Matrices. CATT (errors), Task switching, DV – the overall number of errors. COUNT (errors), Mental counters procedure, DV – the overall number of errors. SST (ssrt), Stop signal task, DV – stop signal reaction time. N-BACK (correct), Figural n-back task, DV – number of correct responses (n = 2). N-BACK (lures), Figural n-back task, DV – number of erroneous responses to lures (n = 1 or n = 3).*

**Table 2** shows the first-order correlation coefficients pertaining to the formerly described variables. Since some DVs were not distributed according to the Gaussian curve, they were log-transformed before entering the correlational procedures. We can see that there are strong inter-correlations between various indices of psychometric SC. In particular, the NAS-50 total score turned out highly correlated with SC Scale (*r* = 0.759), and with the Conscientiousness scale from the NEO-FFI questionnaire (*r* = 0.726). The informant version of our scale (NAS-40) shows much weaker, albeit positive and significant correlations with self-report tools (0.290 < *r* < 0.303, depending on the scale). It seems that assessment provided by peers reveals somewhat different aspects of SC than assessment based on one’s own judgment. **Table 2** also shows that two tests of general fluid intelligence are correlated at the *r* = 0.551 level, which is a result comparable to what has been observed in other studies suing these tools (e.g., Chuderski and Necka, 2012). In contrast to the above-mentioned relationships, the correlation coefficients pertaining to different measures of EFs turned out rather weak, although statistically significant. Some of these correlation coefficients are positive and some are negative because of the nature of dependent variables (the number of errors versus the number of correct responses). Therefore, the absolute value of these coefficients should be taken into account as the strength of relationships. The absolute values oscillate between *r* = 0.069 (n.s.) and *r* = 0.354 (*p* < 0.01). Once again, the mutual relationships between various aspects of executive control appeared not very strong (compare: Duckworth and Kern, 2011; Nęcka et al., 2012). Notably, the absolute values of correlation coefficients between psychometric SC and EFs oscillate between *r* = 0.009 (n.s.) and *r* = 0.128 (*p* < 0.05), and only two of them, out of twenty, surpassed the *p*-level of 0.05.

**Table 2:** Zero-order correlation coefficients between the indices of self-control (NAS-50, NAS-40, SCS, C), general fluid intelligence (TAO, RAPM), and executive functions (CATT, COUNT, SST, N-BACK correct, N-BACK lures).

Variable	NAS-40	SCS	C NEO-FFI	TAO	RAPM	CATT (Errors)	COUNT (Errors)	SST (Ssrt)	N-BACK (Correct)	N-BACK (Lures)
NAS-50	0.290^∗∗^	0.759^∗∗^	0.726^∗∗^	-0.020	0.038	-0.010	0.041	0.013	0.022	0.042
NAS-40		0.303^∗∗^	0.285^∗∗^	0.078	0.096	-0.128*	-0.044	-0.061	0.026	-0.097
SCS			0.682^∗∗^	-0.024	0.022	-0.031	-0.009	-0.079	0.065	-0.030
C NEO-FFI				-0.097	-0.044	0.027	0.115*	-0.071	-0.063	0.021
TAO					0.551**	-0.302**	-0.247**	-0.202**	0.310**	-0.235**
RAPM						-0.270**	-0.275**	-0.116*	0.311**	-0.27**
CATT (errors)							0.233**	0.145**	-0.354**	0.142*
COUNT (errors)								0.069	-0.311**	0.158**
SST (Ssrt)									-0.160**	0.113
										*p* = 0.051
N-BACK (correct)										-0.255**

*NAS-50, Self-control questionnaire, self-report version (Nęcka et al., 2016). NAS-40, Self-control questionnaire, informant version (Nęcka et al., 2016). SCS, Self-Control Scale (Tangney et al., 2004). C (NEO-FFI), Conscientiousness from the NEO-FFI questionnaire. TAO, Analogical Reasoning Test. RAPM, Raven’s Advanced Progressive Matrices. CATT (errors), Task switching, DV – the overall number of errors. COUNT (errors), Mental counters procedure, DV – the overall number of errors. SST (ssrt), Stop signal task, DV – stop signal reaction time. N-BACK (correct), Figural n-back task, DV – number of correct responses (n = 2). N-BACK (lures), Figural n-back task, DV – number of erroneous responses to lures (n = 1 or n = 3). ^∗^p < 0.05 (two-tailed). ^∗∗^p < 0.01 (two-tailed).*

**FIGURE 1:**
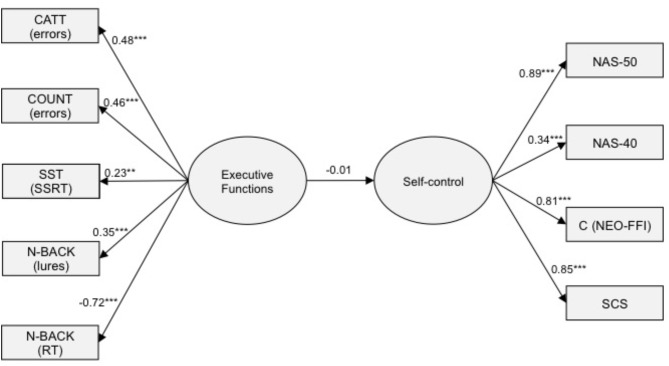
The structural equation model linking executive functions with self-control.

**FIGURE 2:**
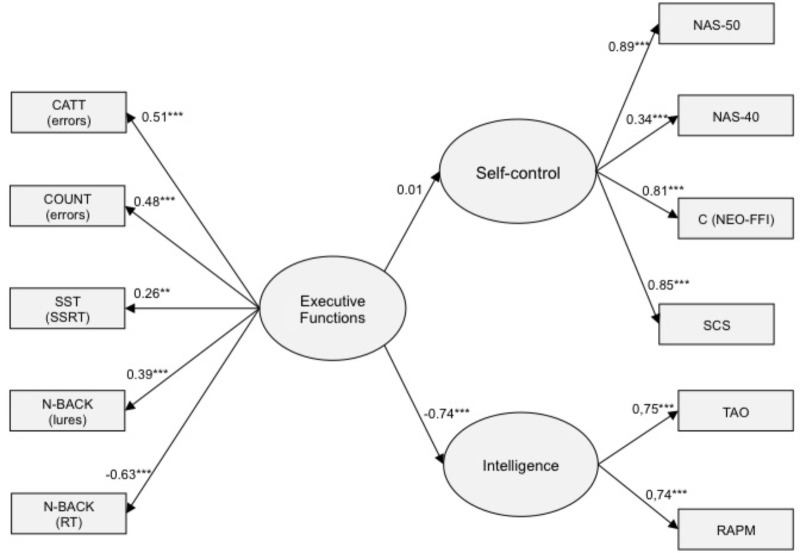
The structural equation model linking executive functions with intelligence and self-control.

In the next step of data analysis, we tested structural models that were supposed to capture the relationships between latent variables. The relationships between SC, fluid intelligence, and EFs were tested by means of latent variable modeling with IBM SPSS Statistics Amos v. 24, using maximum likelihood (ML) estimation method. The latent variable SC was defined by the following measures: NAS50, NAS40, SC Scale and C (NEO-FFI). The latent variable executive functioning was defined by five measures stemming from four tasks: total number of errors for the CATT task, total number of errors for the COUNT task, stop-signal reaction time (SSRT) obtained from the Stop-Signal task, and two measures from the n-back task: the number of correct responses for N2 condition and the number of lures for N1 and N3 conditions. Note that all indicators except of the number of correct responses for N2 condition were reversed: they were either errors or response latencies. Therefore, their higher values indicate lower performance, thus justifying negative signs of relationships reported in **Figures 1, 2**. Finally, the latent variable Fluid intelligence was defined by two tasks: TAO and RAMP. It must be underscored that the interference effect from the Stroop task, computed as the proportion of RT in the incongruent and congruent conditions, did not contribute to this latent variable, since the loading was as low as 0.11. Other DVs obtained from the Stroop task (e.g., latencies, error rates) did not contribute anything, either. For these reasons, we excluded the Stroop task indices from further analyzes, although some models that included Stroop showed acceptable fit.

In the first model, SC was regressed on executive functioning. No error correlations were specified. The analysis revealed evidence for moderate non-normality (skew < 2.7, kurtosis < 7.1) for some measures. This model showed an acceptable model fit: χ^2^(26) = 34.301, *p* = 0.128; CFI = 0.987, RMSEA = 0.033 (90% CI: 0.000, 0.060). **Figure 1** displays the standardized path coefficients of this model. Note that the fit of the measurement model of SC was satisfactory: χ^2^(2) = 0.766, *p* = 0.682; CFI = 1.000, RMSEA = 0.00 (90% CI: 0.00, 0.087). Similarly, the fit of the measurement model of executive functioning was very good: χ^2^(2) = 1.067, *p* = 0.586; CFI = 1.000, RMSEA = 0.00 (90% CI: 0.00, 0.096).

In the next step, both SC and fluid intelligence were regressed on executive functioning (see **Figure 2**). Again, no error correlations were specified. This model confirmed that executive functioning did not predict SC (β = 0.003, n.s.). By contrast, it strongly predicted fluid intelligence (β = –0.74, *p* < 0.001). The correlation between SC and fluid intelligence was set free because zero-order correlation coefficients between SC and intelligence measures turned our very weak (see **Table 2**). The fit of this model was also very good: χ^2^(42) = 50.20, *p* = 0.180; CFI = 0.990, RMSEA = 0.026 (90% CI: 0.00, 0.049). **Figure 2** shows the standardized path coefficients of this model.

We report only the models that obtained acceptable fit indices. Alternative models, built with other DVs, did not fit properly with the data. In particular, models in which the Stroop task was taken into account turned out unacceptable.

## Discussion

In order to examine the significance of EFs for the trait of SC in adult healthy volunteers, we investigated 296 people with the battery of five EF tasks and three psychometric measures of SC. We also added two general fluid intelligence tests (Gf) with the intention to check whether potential relationships between SC and EF would be affected in some way (i.e., strengthened, weakened, mediated) by Gf. In the structural equation modeling approach, we extracted three latent variables, representing executive control, behavioral control, and general fluid intelligence. We found that the EF—SC relationship was non-existent, whereas the EF—Gf relationship turned out quite strong. No relationship between SC and intelligence became evident.

Lack of relationship between the latent variables representing executive control, measured with EF tasks, and psychometric SC, measured with questionnaires, is probably the most important finding of this study. On the one hand, it might be regarded unexpected taking into account the widespread conviction about the importance of EFs for effective control of behavior (e.g., Hofmann et al., 2009, 2012; Diamond and Lee, 2011; Kotabe and Hofmann, 2015). According to this stance, EFs play a crucial role in determination of the efficacy of behavioral SC, being its cognitive substructure. On the other hand, our findings should not be surprising in the context of other studies reporting rather weak relationships of executive control tasks with self-reported measures of behavioral control (e.g., Reynolds et al., 2006; Nęcka et al., 2012). The meta-analysis performed by Duckworth and Kern (2011) seems particular interesting from this point of view because the authors found that the average correlation coefficient between these two types of measures, obtained after examining 282 studies, was as low as *r* = 0.10. What is a tenable explanation of these discrepancies, then?

To begin with, there is a possibility that SC is a personality trait rather than a cognitive ability. Personality traits are believed to be independent of both general intelligence and particular lower-order abilities constituting the *g* factor, although there are arguments that change of research paradigms might reveal still unknown relationships (Ackerman, 2018). According to the mainstream of the personality research, major personality dimensions should be regarded orthogonal to mental abilities. Apart from the existing body of evidence (e.g., McCrae and Costa, 1987; Ackerman and Heggestad, 1997), this conviction may be supported by theoretical arguments. For instance, personality traits are usually bipolar in nature (e.g., extraversion versus introversion) and none of their poles are regarded ‘better’ or ‘worse’ as such. Rather, being close to one of the extremes may help in specific tasks, situations, or job requirements: extraverts usually do better as salespersons although introverts may prevail in laboratory job (Barrick and Mount, 1991). Intellectual traits work in a different manner, since it is usually beneficial for a person to demonstrate high rather than low level of cognitive abilities. General intelligence seems particularly helpful because it contributes to performance in all cognitive tasks. Another argument pertains to the distinction between typical and maximal performance (Goff and Ackerman, 1992). Personality traits shape human behavior in typical, repetitive, everyday situations, whereas intellectual traits determine human performance in very specific situations, such as exams or test taking sessions, in which a person attempts to obtain the best possible result. IQ scores predict real-life achievements with limited precision because of this gap between typical and maximal performance. Standard personality assessment tools (i.e., questionnaires) include items referring to typical everyday situations, whereas standard intellectual tests consist of cognitive tasks that require the highest possible engagement.

If this line of reasoning is sound, we should treat the trait of SC as a dimension belonging to the realm of personality rather than to the category of cognitive abilities. Specifically, this trait probably does not work according to ‘the more the better’ principle, which is characteristic of intellectual abilities. It would be fascinating to reveal possible dark sides of high level of behavioral SC, since over-control may cause a number of problems in social adjustment and personal satisfaction, such as inflexibility or obsessive-compulsive behavior. Anyway, the trait of SC may not need any cognitive functions underlying its mode of functioning. Consequently, it should not enter in any relationship with executive control, measured with EF tasks.

So, it is possible that the trait of SC does not need any underlying cognitive functions but it is also possible that it needs functions that were not investigated in our study. We based this investigation on the Miyake et al. (2000) model of EFs, for its widespread acceptance and popularity. However, this model lacks some EFs that might be important for SC, mostly for its proactive aspects. Careful planning of behavior, including creation a schedule of goals and actions, is undoubtedly an important facet of SC. But planning is rarely taken into account in EF studies, except of some clinical studies in which Shallice (1982) Tower of London (ToL) task is adopted (Mihalec et al., 2017). Although ToL engages short-term planning of actions, which tends to be impaired in the frontal lobe patients, as well as in PD and AD patients, it does not engage the processes involved in long term planning performed by healthy people during their goal-oriented activities. Another EF function that is absent in the Miyake et al. (2000) model pertains to goal maintenance. Inability to remember what is the goal of one’s currently performed action results in chaotic behavior and overly dependence on environmental influences, at the expense of behavior triggered by endogenous decisions. On the contrary, the ability to maintain the goals allows efficient control of actions. Had we included the goal maintenance function into the battery of EF tasks, we might be able to obtain a bit stronger relationships between executive control and behavioral control. Inclusion of updating tasks did not help to resolve this problem because such tasks pertain to short-term updating of the content of working memory rather that long-term keeping in mind personal goals, particularly their hierarchy of importance and time scheduling. Tasks that would be able to engage long-term processes of planning and goal maintenance are still lacking in the standard list of EF procedures, although they seem to be of utmost necessity.

It is also possible that the trait of SC needs EFs, including the ones that were investigated in our study, but we were unable to unveil such relationships due to psychometric reasons. The EF tasks have been designed not for psychometric purposes but for investigation of the general aspects of cognition. Therefore, their psychometric properties are quite low, particularly in reference to stability of measurement. These tasks are also very narrow in scope, meaning that each of them engages a very specific process or function, such as disengagement of attention (the flanker task) or conflict resolution (the Stroop task). For psychometric purposes, the EF tasks should be much broader in scope. Moreover, the existing EF tasks are characterized by large amount of specific variance resulting from specificity of stimuli, procedure, implementation, equipment, instructions, etc. There is no standard rule of construction the EF tasks and their implementation for particular study. Being aware of this problem, we deliberately designed the study in the manner that allowed construction of latent variables, which were supposed to go beyond specificity of various tasks and capture the common variance pertaining to all tasks. To some extent, we succeeded because the latent variable representing executive control demonstrated quite strong relationship with the latent variable representing general fluid intelligence. From this point of view, lack of relationship between EF and the trait of SC turns out to be significant. If the standardized path coefficient between EF and Gf is rather strong, and the analogical coefficient between EF and SC is non-existent, then this ‘negative’ result probably supports the stance according to which SC in adult healthy people does not depend on the strength of executive control. Still, this conclusion must be supplemented with the caveat that different set of EF tasks might have resulted in quite different pattern of relationships between the latent variables.

Another explanation of the lack of any EF—SC relationship pertains to the characteristics of the sample. We investigated healthy adult volunteers who demonstrated the wide range of the trait of SC, whereas studies demonstrating the existence of the EF—SC relation were typically run with special populations, such as incarcerated violent offenders (Seruca and Silva, 2016; Meijers et al., 2017). Still, the relationships reported are rather weak. For instance, Meijers et al. (2017) investigated 130 prisoners with a neuropsychological battery suitable to assess such functions as response inhibition, planning, attention, shifting, working memory, and impulsivity. They found only one significant difference between violent and non-violent offenders, which referred to response inhibition (partial correlation *r* = 0.205). They also report a weak relationship between recidivism and planning (*r* = 0.209). As we can see, some EFs may demonstrate predictive value for SC when the latter is really weak. If the whole range of SC variance is taken into account, such relationships – being generally scarce and weak – disappear. Interestingly, the evidence demonstrating the predictive value of SC for various aspects of life success pertains mostly to low level of this trait, so to speak – to lack of SC. For instance, the results reported by Moffitt et al. (2011) show that it is the low level of SC that predicts such teenage problems as smoking, school absenteeism, or unplanned parenthood. Their participants were divided into quintiles according the informant-based ratings of SC. The first quintile differed substantially from the rest of participants, while the fifth quintile – representing to highest level of SC – did not contribute much as to prediction of behavioral conduct or misconduct. It is possible, then, that SC is important for life success in the sense that lack of it predicts many problems but its high level of development does not have predictive value anymore. In other words, there may be a threshold principle involved in this relationship: the trait of SC might be important up to some specific value (threshold), above which it loses significance as a predictor of life success.

Finally, there is a possibility that self-report measures do not provide exact estimation of the individual capacity to control one’s behavior. Consequently, they should be replaced with some more objective measures, such as informant reports (e.g., Moffitt et al., 2011) or specially devised experimental tasks(e.g., Steimke et al., 2016). SC is a highly valued personal trait, therefore the social desirability factor is likely to influence the way in which people approach particular items in self-report questionnaires. Deliberate decision to present oneself in a positive way is probably not very likely in procedures that assure full anonymity, as was the case of the present study. Still, at least some participants could choose to present themselves as more ‘organized’ and ‘reliable’ than they know is the case in reality. Moreover, the results could be biased not only due to conscious decisions to boost the questionnaire results but also because of reduced awareness one’s own personal traits. We simply may not know how much control do we have over our own cognitive control (Nęcka et al., 2012), therefore, our questionnaire responses may not reveal the real state of affairs. However, this kind of bias seems unlikely to act in just one direction, namely, toward the unrealistically high level of assessment. If people are not aware how much control do they have, they may either overestimate or underestimate their capability of behavioral control. In consequence, the overall results should not be systematically heightened, although reliability of assessment is likely to suffer. To prevent this threat, items of our questionnaires did not require general knowledge about one’s trait but only some level of awareness concerning specific situations. For instance, we did not ask ‘Do you think you are a self-controlling person?’. Rather, we attempted to ask, for instance, about being late for meetings or doing deadlines. Additionally, we supplemented the battery of SC tools with the informant-based questionnaire NAS-40. Still, there is a possibility that the battery of tools supposed to assess the trait of SC suffered from subjectivity and bias toward social desirability.

This study suffers from some limitations that make the final conclusions questionable. Firstly, the number and variety of EFs tasks should be increased. EFs responsible for planning and goal maintenance seem particularly important for SC but they are mostly missing in experimental studies, including ours. Working memory updating tasks appear to involve short-term goal maintenance, but not planning. Secondly, assessment of SC should be made more objective, for instance through application of observational scales referring to participants’ behavioral characteristics (e.g., Moffitt et al., 2011). We used the informant version of the SC questionnaire, which undoubtedly helped to improve objectivity of assessment, but this solution is far from perfect, mostly because of limited knowledge the informants may have concerning the ‘real’ level of SC represented by the participants proper. The objective measures of SC are rather difficult to employ because of the very nature of SC, which seems to be a multi-dimensional and multi-faceted phenomenon. Self-report questionnaires, in spite of all their limitations, have a fundamental advantage: they allow holistic and generalized assessment that goes beyond specific situations and specific impairments. Still, a balanced combination of self-reported and objective sources of knowledge should be adopted in further studies. Finally, our sample of participants, albeit quite large, was probably not diversified enough concerning age, socio-economic status, and the general level of the trait of SC. In particular, we lacked participants who would suffer from mild, sub-clinical impairments of SC. Maybe the relationship we were not able to find takes place only as far as such people are concerned.

## Conclusion

While planning this study, we assumed that at least weak relationships between the trait of SC and efficiency of executive control would turn out significant. Former studies were conducted with smaller samples and usually without latent variable modeling. Since latent variables go beyond specific variance produced by particular measurement tasks and procedures, thus capturing ‘the essence’ of the constructs of interest, such modeling seemed much more promising than regular correlational approach. So far, the hypothesis that EFs constitute the cognitive substrate of the trait of SC must be rejected. In its strong version, our take-home message would sound like the following: EFs are not significant for SC, probably because they belong to the realm of abilities whereas the latter is a part the personality domain. In a weak and humble version, the message is that we were not able to prove such a relationship.

## Author Contributions

EN designed the study and drafted the manuscript. AG helped to prepare the materials, performed all major statistical analyses, and helped to prepare the final version of the manuscript. JO helped to prepare the materials and improved the final version of the manuscript. MN and NW helped to prepare the materials, participated in the data gathering, and helped to prepare the final version of the manuscript.

## Conflict of Interest Statement

The authors declare that the research was conducted in the absence of any commercial or financial relationships that could be construed as a potential conflict of interest.
